# Change of Hemoglobin Levels in the Early Post-cardiac Arrest Phase Is Associated With Outcome

**DOI:** 10.3389/fmed.2021.639803

**Published:** 2021-06-09

**Authors:** Christoph Schriefl, Christian Schoergenhofer, Florian Ettl, Michael Poppe, Christian Clodi, Matthias Mueller, Juergen Grafeneder, Bernd Jilma, Ingrid Anna Maria Magnet, Nina Buchtele, Magdalena Sophie Boegl, Michael Holzer, Fritz Sterz, Michael Schwameis

**Affiliations:** ^1^Department of Emergency Medicine, Medical University of Vienna, Vienna, Austria; ^2^Department of Clinical Pharmacology, Medical University of Vienna, Vienna, Austria; ^3^Department of Medicine I, Medical University of Vienna, Vienna, Austria

**Keywords:** critical care, cardiac arrest, post-cardiac arrest syndrome, hemoglobin, resuscitation, mortality, neurologic outcome, vascular permeability

## Abstract

**Background:** The post-cardiac arrest (CA) phase is characterized by high fluid requirements, endothelial activation and increased vascular permeability. Erythrocytes are large cells and may not leave circulation despite massive capillary leak. We hypothesized that dynamic changes in hemoglobin concentrations may reflect the degree of vascular permeability and may be associated with neurologic function after CA.

**Methods:** We included patients ≥18 years, who suffered a non-traumatic CA between 2013 and 2018 from the prospective Vienna Clinical Cardiac Arrest Registry. Patients without return of spontaneous circulation (ROSC), with extracorporeal life support, with any form of bleeding, undergoing surgery, receiving transfusions, without targeted temperature management or with incomplete datasets for multivariable analysis were excluded. The primary outcome was neurologic function at day 30 assessed by the Cerebral Performance Category scale. Differences of hemoglobin concentrations at admission and 12 h after ROSC were calculated and associations with neurologic function were investigated by uni- and multivariable logistic regression.

**Results:** Two hundred and seventy-five patients were eligible for analysis of which 143 (52%) had poor neurologic function. For every g/dl increase in hemoglobin from admission to 12 h the odds of poor neurologic function increased by 26% (crude OR 1.26, 1.07–1.49, *p* = 0.006). The effect remained unchanged after adjustment for fluid balance and traditional prognostication markers (adjusted OR 1.27, 1.05–1.54, *p* = 0.014).

**Conclusion:** Increasing hemoglobin levels in spite of a positive fluid balance may serve as a surrogate parameter of vascular permeability and are associated with poor neurologic function in the early post-cardiac arrest period.

## Introduction

Low hemoglobin (Hb) levels have been associated with poor clinical outcome in cardiac arrest patients. Anemia is common in the post-resuscitation period and, conceivably, the oxygen-transport capacity of Hb may be especially important during and after global hypoxia. Previous studies therefore focused on mean Hb levels either on admission or in the first days after cardiac arrest and its associations with clinical outcomes, but the kinetics of Hb after successful resuscitation and their clinical significance remain unknown (1–5).

During the last decades the post-cardiac arrest syndrome (PCAS) has been increasingly understood as a sepsis-like condition. Comparable to sepsis, patients after cardiac arrest may display dysregulated inflammation, myocardial and adrenal dysfunction, coagulopathy and a disrupted endothelial barrier function indicating similar pathomechanisms (6, 7). However, especially the latter demands further investigation. Capillary leak is characterized by increased endothelial permeability leading to a consecutive loss of proteins and fluid from the intravascular to the interstitial space (8–12). Biomarkers such as angiopoietin-2, vascular endothelial growth factor and soluble fms-like tyrosine kinase 1, have been suggested as indicative for endothelial permeability and were likewise associated with mortality in septic patients (13), but may lack specificity and are mostly not available during routine care.

Still, extravascular fluid loss due to lacking endothelial barrier function challenges treating physicians as optimal fluid resuscitation and vasopressor support are required to provide adequate cardiac output and consecutive organ perfusion (14). However, the extent of extravascular fluid loss remains difficult to determine *in vivo*. With regards to PCAS, oedema formation, especially in the brain, is a crucial factor for neurologic outcome (15, 16).

Similar to PCAS, vascular hyperpermeability has also been described in other critical conditions such as severe burn injury (17, 18). In a burn injury animal model, hematocrit increased continuously in the first hours after the injury indicating the loss of fluid to the extracellular space and a relative increase of red blood cells (19).

In this context, we hypothesized that Hb kinetics may reflect extravascular fluid losses. Due to their large size, erythrocytes, and therefore Hb, remain in the circulation. As a consequence of the increased vascular permeability, Hb concentrations increase, similar to capillary leak syndrome, in which values >20 g/dL may occur, even in spite of intravenous fluid administration (9, 10).

In this study we investigated dynamic changes in Hb concentrations as an indicator of vascular permeability in the early post-cardiac arrest phase and its association with neurologic outcome.

## Methods

### Study Design

We analyzed prospectively collected data from the Vienna Clinical Cardiac Arrest Registry of the Department of Emergency Medicine at the Medical University of Vienna, a tertiary care facility. The registry includes all adult cardiac arrest patients, admitted to, and treated at the Department of Emergency Medicine.

Data acquisition and documentation was conducted in accordance with the Utstein style recommendations for cardiac arrest related documentation (20) and has recently been described in detail for our registry (21).

Blood samples were drawn immediately after admission and analyzed by the ISO-certified central laboratory of the Vienna General Hospital. The first blood sample always includes hemoglobin (g/dL) as a standard hematological parameter. Further blood samples including hematological variables were routinely drawn every 6 h after ROSC for the first day. Total intravenous fluid, fluid balance, total urine volume and catecholamine doses were documented after 12 and 24 h in health care records as a routine documentation. This study complies with the declaration of Helsinki and was approved by the local Ethics Committee of the Medical University of Vienna (EK No. 1219/2018).

### Study Population

All adults ≥18 years of age with a non-traumatic cardiac arrest (out-of hospital and in-hospital cardiac arrest) between January 2013 and December 2018 were included. Patients (i) without return of spontaneous circulation, (ii) with extracorporeal cardiopulmonary resuscitation (eCPR) therapy, (iii) immediately regaining consciousness, (iv) who died within 24 h after ROSC, (v) undergoing surgery within 24 h after ROSC, (vi) requiring blood transfusions within 24 h after ROSC, (vii) with bleeding (any form of bleeding within 24 h documented in the patient charts or transfer reports), and (viii) without targeted temperature management (TTM) were excluded. Furthermore, only patients with full datasets for multivariable analysis were eligible.

Patient treatment was based on current guidelines, including post-resuscitation management (14). TTM (32–34°C) was conducted as soon as possible (via surface or intravascular cooling) for all comatose patients according to an institutional protocol based on the current guidelines within 60 min after admission. TTM was maintained for 24 h until the start of rewarming. Rewarming was performed with 0.25°C/h with a maximum rate of 0.5°C/h. As all patients in our cohort were treated according to the institutional protocol, all analyses and blood sampling were performed within the TTM phase.

**Figure 1:**
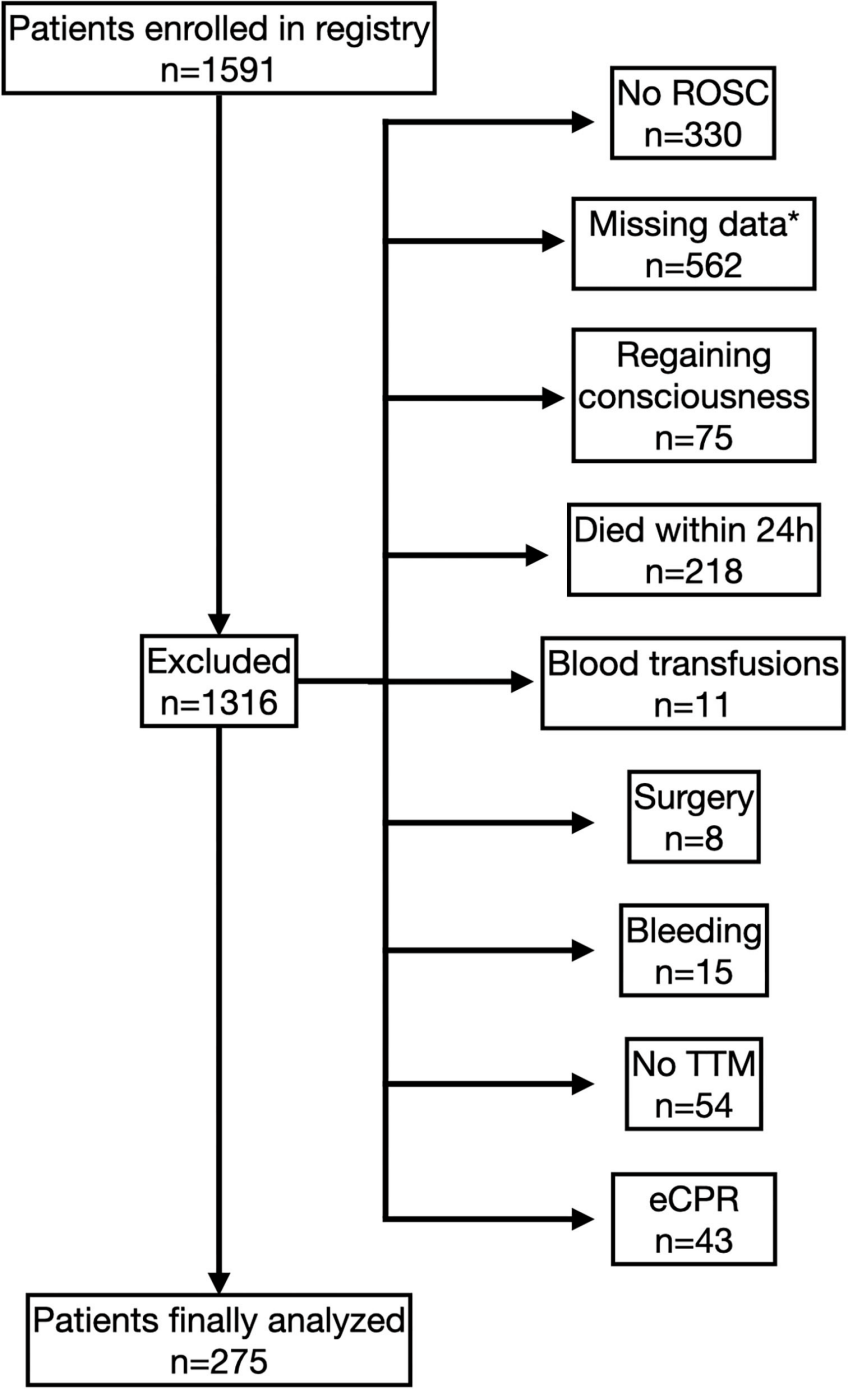
Study flow chart. Flow diagram of enrolment and exclusion of patients. *Only patients with full datasets for multivariable analyses were eligible.

During the observation period 1,591 cardiac arrest patients were enrolled in our registry. Of those patients, 275 fulfilled the inclusion criteria and were finally analyzed (Figure 1).

### Endpoints

The primary endpoint was neurologic function at day 30, defined according to the Cerebral Performance Category (CPC) scale: good neurologic outcome was defined as a CPC 1 (good cerebral performance) or 2 (moderate disability); poor neurologic outcome was defined as CPC 3–5 (severe disability, vegetative state, or death) or persistent unresponsiveness due to analgosedation during the study period or before death. The choice of this composite endpoint is in accordance with the Utstein recommendations (20).

Secondary endpoints included 30-day mortality and exploratory correlations between Hb kinetics, time intervals of resuscitation, fluid balance, noradrenaline (norepinephrine) dose and pH value.

### Statistical Analysis

We present categorized data as counts (and relative frequency) and scale data as medians with 25–75% interquartile range (IQR). We calculated differences of Hb (dHb; Hb at 12 h–Hb at admission = dHb12h; Hb at 24 h–Hb at admission = dHb24h). For exploratory analysis of selected baseline variables and for the comparison of Hb differences between patients with good and poor neurologic function at day 30 the Mann-Whitney *U*-test or the Chi^2^-test were applied, as applicable. We performed receiver operating characteristic (ROC) curve analysis to determine (i) the optimal timepoint for this analysis (12 or 24 h after ROSC) and (ii) the optimal cut-off of Hb differences between the chosen time-points. Results are given as area under the curve (AUC) with 95 % confidence intervals (95% CI) and *p*-value. According to this cut-off we dichotomized the patients into two groups. Selected baseline data were also compared between the two derived groups in an exploratory fashion, as explained above. Furthermore, we subdivided the sample into dHb quartiles. Percentages of patients with poor and good neurologic outcome at day 30 according to dHb12h quartiles are presented as table and plot.

We used a binary logistic regression analysis (backward stepwise elimination approach according to Wald test-statistics step-by-step) to estimate the effect of the respective dHb (as continuous variable and as categories) on the primary endpoint. The effect was quantified as odds ratio (OR) with 95 % confidence intervals (95% CI).

We used a Cox regression analysis (backward stepwise elimination approach according to Wald test-statistics step-by-step) to estimate the effect of dHb on the secondary endpoint taking the time-to-event into account. The effect was quantified as hazard ratio (HR) with 95 % confidence intervals (95% CI).

We selected covariables for the multivariable models based on previous studies (22–27). These variables included age (years), sex, initial heart rhythm at the scene (shockable vs. non-shockable), basic life support (BLS), witness status, number of shocks applied, cumulative adrenaline (epinephrine) dose (mg) during resuscitation and pH on admission. Furthermore we included noradrenaline (norepinephrine) dose and fluid balance of the first 12 h after ROSC in the analysis, because both therapies are used to ensure hemodynamic stability and fluid therapy might impact on the main parameter of interest: the Hb kinetics. The final results of the multivariable analyses are presented in tables. Those variables eliminated by the testing procedure were omitted. All steps of the logistic regression analysis (backward stepwise elimination approach according to Wald test-statistics step-by-step) for the primary endpoint are presented in the Supplementary Tables 9, 10.

We performed sensitivity analyses for the primary outcome including only patients with out-of-hospital cardiac arrest, with patients receiving BLS and with patients not receiving BLS. Furthermore we included C-reactive protein concentrations and white blood cell counts into an auxiliary multivariable analysis (Supplementary Tables 7, 8). Additionally, we modeled the expected decrease in hemoglobin based on estimated plasma volume and overall fluid balance. The difference between expected and real hemoglobin was calculated and included in the analysis as an additional sensitivity analysis (refer to the Supplementary Material for further details).

Kaplan-Meier plots of the estimated 30-day mortality were conducted for each Hb category and compared using the log-rank test. Exploratory correlations were calculated by applying the non-parametric Spearman procedure.

For data management and analyses we used IBM SPSS Statistics (Version 26, IBM Corporation) and R (R Foundation for Statistical Computing, Version 3.6.2). A two-sided *p*-value < 0.05 was considered statistically significant.

## Results

**Figure 2:**
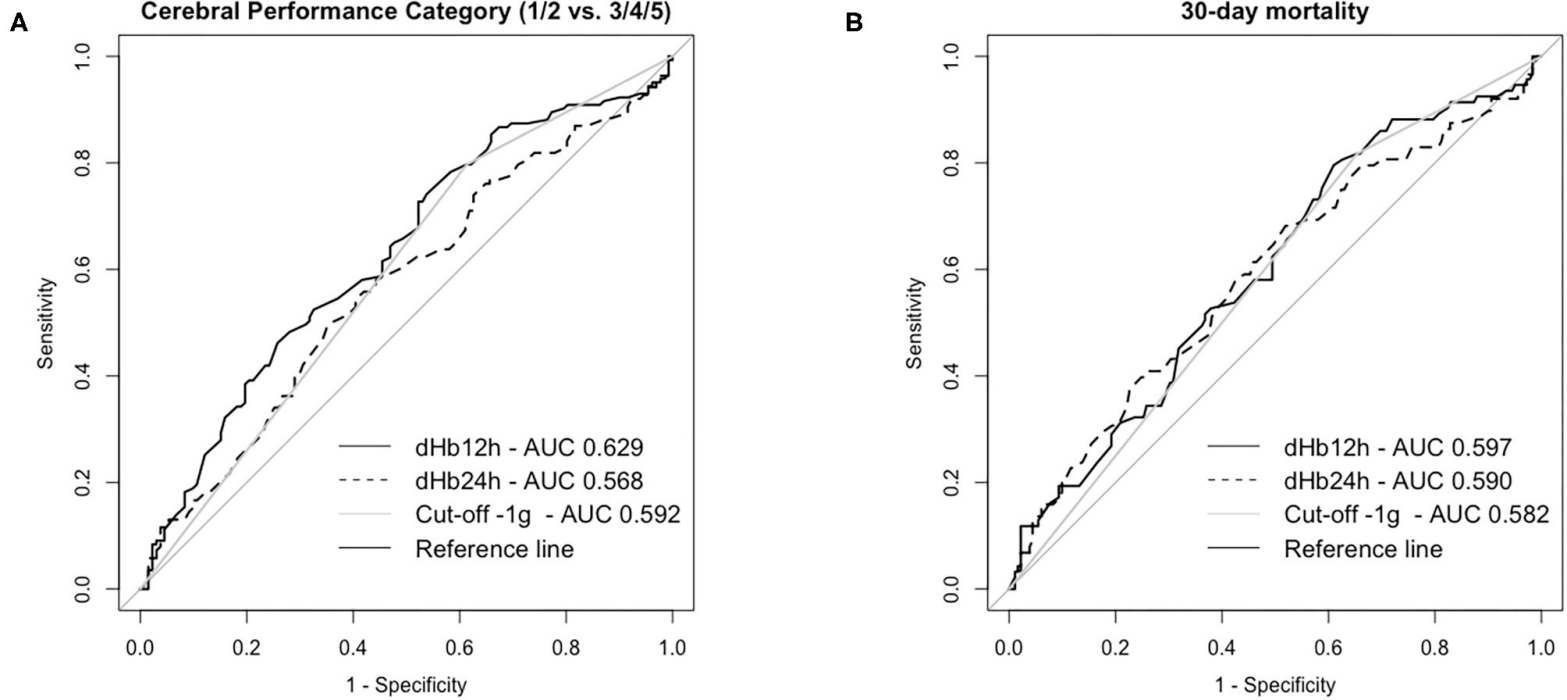
Receiver Operating Characteristic Curves for neurologic outcome **(A)** and mortality **(B)** at day 30 in patients successfully resuscitated from cardiac arrest. dHb12h = Hemoglobin (Hb) levels 12 h after return of spontaneous circulation (ROSC)—Hb levels on admission; dHb24h = Hb levels 24 h after ROSC—Hb levels on admission; Cut-off−1g: categorized data for patients with </≥-1 g difference for Hb levels after 12 h—Hb levels on admission.

In ROC analysis, the AUC of dHb12h was 0.62 (95% CI 0.56–0.69, *p* < 0.001 compared to the reference line) and for dHb24h 0.57 (95% CI 0.50–0.64, *p* = 0.054) for neurologic outcome (Figure 2). We therefore decided to use dHb12h for our analysis.

There was no obvious cut-off identifiable in ROC analysis for neurologic outcome (Figure 2 and Supplementary Material). Subsequently, we performed ROC analysis for 30-day mortality (AUC of dHb12h 0.60, 95% CI, 0.53–0.67, *p* = 0.007). Based on the results of both analyses, we decided to use a difference of −1 g/dL Hb (AUC 0.59, 95% CI 0.52–0.66, *p* = 0.008 for neurologic outcome and AUC 0.58, 95% CI 0.51–0.65, *p* = 0.021 for 30-day mortality) as a cut-off since this cut-off is (i) practicable for clinicians and (ii) shows acceptable sensitivity of ~80% and a specificity of ~40% for neurologic outcome and 30-day mortality. We dichotomized patients according to their dHb12h into two groups: dHb12h > −1 g/dL = “Group A” (equals a decrease in Hb concentrations of >1 g/dL in 12 h) and dHb12h ≤ −1 g/dL = “Group B” (equals any increase in Hb or a decrease of <1 g/dL in 12 h).

### Baseline Characteristics

Table 1 shows the baseline characteristics of the study population, including distribution of cardiac arrest related parameters, according to 30-day neurologic outcome. Patients with poor neurologic outcome were older (median 63 vs. 55 years, *p* < 0.001) and presented less often with an initial shockable rhythm (41 vs. 80%, *p* < 0.001) or a cardiac cause of cardiac arrest (62 vs. 79%, *p* < 0.001). In addition, they had higher blood lactate levels (7.8 vs. 5.5 mmol/L, *p* < 0.001), lower pH values (7.12 vs. 7.22, *p* < 0.001), a greater, positive fluid balance (2.15 vs. 1.60 L, *p* = 0.002) and lower Hb levels at admission (13.3 vs. 14.1 g/dL, *p* < 0.001).

**Table 1:** Baseline characteristics by neurologic outcome.

	**All**	**Good neurologic outcome**	**Poor neurologic outcome**
	***n* = 275**	***n* = 132**	***n* = 143**
Age, median (IQR)	60 (50–71)	55 (47–67)	63 (53–74)
Female, *n* (%)	75 (27)	32 (24)	43 (30)
Height (cm), median (IQR)	175 (170–180)	180 (170–185)	175 (168–180)
Weight (kg), median (IQR)	85 (75–95)	85 (75–100)	80 (70–95)
Chronic health conditions, *n* (%)			
Diabetes	55 (20)	24 (18)	31 (22)
Hypertension	114 (41)	40 (32)	74 (52)
Current smoker	77 (28)	39 (29)	38 (19)
Chronic heart failure	24 (9)	7 (6)	17 (12)
Myocardial infarction	37 (13)	14 (11)	23 (16)
Cerebral vascular insufficiency	13 (5)	4 (5)	9 (6)
Coronary artery disease	55 (20)	22 (18)	33 (23)
Chronic obstructive pulmonary disease	37 (13)	9 (7)	28 (20)
Pre-arrest CPC 1/2, *n* (%)	275 (100)	132 (100)	143 (100)
Out of hospital cardiac arrest, *n* (%)	255 (93)	124 (94)	131 (92)
Witnessed, *n* (%)	232 (84)	117 (89)	115 (80)
BLS, *n* (%)	175 (64)	90 (68)	85 (59)
Initial shockable rhythm, *n* (%)	164 (60)	105 (80)	59 (41)
No flow (min), median (IQR)^a^	0 (0–1)	0 (0–1)	0 (0–1)
Low flow (min), median (IQR)^a^	22 (14–35)	17 (12–27)	26 (19–38)
Origin, *n* (%)			
Pulmonary	37 (13)	9 (67	28 (20)
Cardiac	186 (68)	104 (79)	82 (62)
Metabolic	5 (2)	1 (1)	4 (3)
Intoxication	8 (3)	2 (2)	6 (4)
Drowning	4 (1)	1 (1)	3 (2)
Sepsis	1 (0.4)	0 (0)	1 (1)
Cerebral	6 (2)	1 (1)	5 (3)
Other	6 (2)	4 (3)	2 (1)
Unknown	22 (8)	10 (8)	12 (8)
Total dose of adrenaline in mg, median (IQR)	2 (0–3)	1 (0–2)	3 (1–4)
Number of shocks applied, median (IQR)^b^	2 (0–4)	2 (1–4)	1 (0–4)
pH, median (IQR)	7.16 (7.05–7.27)	7.22 (7.12–7.28)	7.12 (7.0–7.22)
Lactate (mmol/l), median (IQR)	6.7 (4.3–9.7)	5.5 (3.4–8.4)	7.8 (5.7–10.6)
Hb at admission (g/dL), median (IQR)	13.7 (12.4–14.7)	14.1 (13.0–15.0)	13.3 (11.9–14.3)
Volume (ml/12 h), median (IQR)	2,950 (2,170–3,850)	2,900 (2,150–3,650)	3,030 (2,200–4,000)
Diuresis (ml/12 h), median (IQR)	900 (500–1,530)	1,130 (603–1,700)	750 (370–1,300)
Fluid balance (ml/12 h), median (IQR)	1,900 (1,000–2,700)	1,600 (860–2,350)	2,150 (1,200–3,000)
Noradrenaline dose (μg/kg/min), median (IQR)	0.1 (0.05–0.23)	0.08 (0.04–0.17)	0.15 (0.07–0.33)
Hb change 12 h (g/dl), median (IQR)	−0.1 (−1.1 to 0.8)	−0.5 (−1.4 to 0.4)	0.2 (−0.8 to 1)
CPC 1/2, *n* (%)	132 (48)	132 (100)	0 (0)
Mortality day 30, *n* (%)	93 (34)	0 (0)	93 (65)

*Good neurologic outcome was defined as Cerebral Performance Category 1 to 2, poor neurologic outcome as Cerebral Performance Category 3–5*.

*No flow = time from collapse to the start of resuscitation efforts; Low flow = time is the interval between the start of resuscitation efforts and ROSC; Volume = total volume of intravenous fluids administered within 12 h; Diuresis = urine output from admission to 12 h; Fluid balance = difference between the total volume of intravenous fluids administered and the urine output at 12 h*.

*BLS, basic life support; CPC, Cerebral Performance Category; Hb, hemoglobin*.

a *Data only for witnessed available*.

b *Data only from patients receiving at least one shock*.

Baseline data and demographics of patients subdivided according to dHb12h also differed slightly (Table 2). Patients in group A were younger (58 vs. 62 years, *p* = 0.044) and had a higher percentage of cardiac cause of cardiac arrest (80 vs. 63% *p* = 0.005). The number of patients with an initial shockable rhythm (70 vs. 55%), lactate levels (6.8 vs. 6.7 mmol/L), pH levels (7.16 vs. 7.16), and fluid balance (1.95 vs. 1.88 L) did not differ significantly between groups.

**Table 2:** Baseline characteristics according to dHb12h group.

	**All**	**Group A**	**Group B**
	***n* = 275**	***n* = 80**	***n* = 195**
Age, median (IQR)	60 (50–71)	58 (47–66.5)	62 (50–72)
Female, *n* (%)	75 (27)	20 (25)	55 (28)
Height (cm), median (IQR)	175 (170–180)	176.5 (170–181)	175 (170–180)
Weight (kg), median (IQR)	85 (75–95)	85 (75–91)	85 (75–100)
Chronic health conditions, *n* (%)			
Diabetes	55 (20)	16 (20)	39 (20)
Hypertension	114 (41)	26 (33)	88 (45)
Current smoker	77 (28)	23 (29)	54 (28)
Chronic heart failure	24 (9)	7 (9)	17 (9)
Myocardial infarction	37 (13)	11 (14)	26 (13)
Cerebral vascular insufficiency	13 (5)	4 (5)	9 (5)
Coronary artery disease	55 (20)	15 (19)	40 (21)
Chronic obstructive pulmonary disease	37 (13)	7 (9)	30 (15)
Pre-arrest CPC 1/2, *n* (%)	275 (100)	80 (100)	195 (100)
Out of hospital cardiac arrest, *n* (%)	255 (93)	78 (98)	177 (91)
Witnessed, *n* (%)	232 (84)	68 (85)	164 (84)
BLS, *n* (%)	175 (64)	58 (73)	117 (60)
Initial shockable rhythm, *n* (%)	164 (60)	56 (70)	108 (55)
No flow (min), median (IQR)^a^	0 (0–1)	0 (0–0)	0 (0–1)
Low flow (min), median (IQR)^a^	22 (14–35)	21.5 (14–36)	23 (14–34)
Origin, *n* (%)			
Pulmonary	37 (13)	7 (9)	30 (15)
Cardiac	186 (68)	64 (80)	122 (63)
Metabolic	5 (2)	2 (3)	3 (2)
Intoxication	8 (3)	1 (1)	7 (4)
Drowning	4 (1)	2 (2)	2 (1)
Sepsis	1 (0.4)	0 (0)	1 (1)
Cerebral	6 (2)	2 (3)	4 (2)
Other	6 (2)	0 (0)	6 (3)
Unknown	22 (8)	2 (3)	20 (10)
Total dose of adrenaline in mg, median (IQR)	2 (0–3)	2 (0–3.5)	2 (0.5–3)
Number of shocks applied, median (IQR)^b^	2 (0–4)	2 (1–4)	1 (0–4)
pH, median (IQR)	7.16 (7.05–7.27)	7.16 (7.05–7.26)	7.16 (7.06–7.27)
Lactate (mmol/l), median (IQR)	6.7 (4.3-9.7)	6.8 (4.5-9.3)	6.7 (4.2-9.7)
Hb at admission (g/dL), median (IQR)	13.7 (12.4–14.7)	14.2 (13.3–15.2)	13.4 (12.2–14.6)
Volume (ml/12 hrs), median (IQR)	2,950 (2,170–3,850)	3,015 (2,200–4,000)	2,900 (2,150–3,750)
Diuresis (ml/12 h), median (IQR)	900 (500–1,530)	1,075 (565–1,600)	800 (450–1,500)
Fluid balance (ml/12 h), median (IQR)	1,900 (1,000–2,700)	1,949 (1,000–2,800)	1,875 (1,010–2,650)
Noradrenaline dose (μg/kg/min), median (IQR)	0.1 (0.05–0.23)	0.09 (0.05–0.18)	0.12 (0.06–0-29)
Hb change 12 h (g/dl), median (IQR)	−0.1 (−1.1 to 0.8)	−1.6 (−2.2 to 1.3)	0.4 (−0.2 to 1)
CPC 1/2, *n* (%)	132 (48)	51 (64)	81 (42)
Mortality day 30, *n* (%)	93 (34)	17 (21)	76 (39)

*Group A = a decrease in hemoglobin (Hb) >1 g/dL within 12 h after return of spontaneous circulation (ROSC); group B = increase in Hb levels or a decrease of ≤ 1 g/dL Hb within 12 h after ROSC*.

*No flow = time from collapse to the start of resuscitation efforts; Low flow = time is the interval between the start of resuscitation efforts and ROSC; Volume = total volume of intravenous fluids administered within 12 h; Diuresis = urine output from admission to 12; Fluid balance = difference between the total volume of intravenous fluids administered and the urine output at 12 h*.

*BLS, basic life support; CPC, Cerebral Performance Category; Hb, hemoglobin*.

a *Data only for witnessed available*.

b *Data only from patients receiving at least one shock*.

### Outcome Analysis

DHb12h differed significantly between patients with good or poor neurologic outcome [−0.5 (IQR: −1.4–0.4) vs. 0.2 (IQR: −0.8–1) g/dL, *p* < 0.001, Table 1] and 30-day mortality [−0.3 (IQR: −1.3–0.7) vs. 0.2 (IQR −0.7–0.9) g/dL, *p* = 0.009].

#### 30-Day Neurologic Function

In univariate analysis, the crude OR for dHb12h was 1.26 (95% CI 1.07–1.49, *p* = 0.006) to have poor neurologic outcome. After multivariable adjustment for age, sex, initial rhythm, BLS, witness status, number of shocks, cumulative adrenaline dose, pH on admission, fluid balance and noradrenaline dose the adjusted OR of dHb12h was 1.27 (95% CI 1.05–1.54, *p* = 0.014) to have a poor neurologic outcome (Table 3). These results correspond to an approximate 26%, respectively 27%, increased risk of poor outcome for every g/dL increase in Hb concentrations within the first 12 h after ROSC.

**Table 3:** Multivariable analysis of the primary endpoint.

	**Poor outcome (Cerebral Performance Category 3–5)**			
**Variables**	**OR (95% CI)**	***p*-value**	**OR (95% CI)**	***p*-value**
dHb12h (g/dl)	1.27 (1.05–1.54)	0.014	-	
dHb12h group	-		2.69 (1.42–5.10)	0.002
Age (years)	1.04 (1.02–1.06)	<0.001	1.04 (1.02–1.06)	<0.001
Number of shocks applied	0.82 (0.74–0.91)	<0.001	0.83 (0.75–0.92)	<0.001
Total dose of adrenaline (mg)	1.55 (1.28–1.87)	<0.001	1.54 (1.28–1.85)	<0.001
pH value	0.11 (0.01–0.93)	0.042	0.09 (0.01–0.76)	0.027
Fluid balance 12 h (L)	1.23 (0.99–1.53)	0.067	1.23 (0.99–1.54)	0.062

*Poor outcome was analyzed by binary logistic regression using a backward stepwise elimination approach according to Wald test statistic step-by-step. The presented variables are the ones remaining in the final step of the model*.

*dHb12h (g/dl) = Hemoglobin (Hb) levels 12 h after return of spontaneous circulation (ROSC)—Hb levels on admission. dHb12h group = group A (equals a decrease in Hb concentrations of >1 g/dL in 12 h after ROSC) vs. group B (equals any increase in Hb or a decrease of ≤ 1 g/dL in 12 h after ROSC)*.

*CI, confidence interval; Hb, hemoglobin; OR, odds ratio*.

In univariate analysis, the crude OR for group B was 2.48 (95% CI 1.45–4.24, *p* < 0.001) to have a poor neurologic outcome. After multivariable adjustment for the above-mentioned covariates the adjusted OR was 2.69 (95% CI 1.42–5.10, *p* = 0.002) to have a poor neurologic outcome (Table 3). Patients in group B have an ~2.5-fold, respectively, 2.7-fold, increased risk to have poor neurologic outcome after cardiac arrest.

We performed sensitivity analyses in patients who received BLS (*n* =175) or not (*n* = 100) and in patients with out-of-hospital cardiac arrest only (*n* = 255), which are presented in detail in the Supplementary Material. In short, we obtained similar results in the population of patients with out-of-hospital cardiac arrest.

In the population of patients who received BLS, uni- and multivariable analyses of the continuous variable were non-significant, while the dichotomized variable was found to be significantly associated with neurologic outcome with an odds ratio of 2.38 (95% CI, 1.24–4.56, *p* = 0.009) in univariate analysis and 2.41 (95% CI, 1.11–5.25, *p* = 0.027) in multivariable analyses. In contrast to that, in patients who did not receive BLS (*n* = 100), effect sizes were larger [e.g., univariate analysis of dHb12h 1.50 (1.12–2.0, *p* = 0.006) and multivariable analysis 1.5 (1.09–2.07, *p* = 0.013)] and results were statistically significant despite the smaller sample size.

Furthermore, in another sensitivity analysis, we investigated whether the inclusion of inflammatory biomarkers C-reactive protein and white blood cell counts affected the model (Supplementary Tables 7, 8). In short, admission C-reactive protein concentrations and admission blood cell counts were eliminated from the model and effect sizes remained stable with an adjusted OR of dHb12h of 1.28 (95%CI, 1.05–1.56, *p* = 0.014). However, when we included C-reactive protein concentrations and white blood cell counts at 48 h both parameters remained in the model. The effect size of dHb12h was not relevantly altered with an adjusted OR of dHb12h of 1.22 (95%CI, 1.01–1.47, *p* = 0.044).

#### 30-Day Mortality

In univariate analysis, the crude HR of dHb12h for 30-day mortality was 1.19 (95% CI 1.04–1.36, *p* = 0.011). After multivariable adjustment for the above-mentioned covariates, the estimates of dHb12h on 30-day mortality in the adjusted model showed a HR of 1.16 (95% CI 1.02–1.31, *p* = 0.025) (Table 4). These results correspond to an approximate 19%, respectively 16%, increased risk for 30-day mortality for every g/dL increase in Hb concentrations within the first 12 h after ROSC.

**Table 4:** Multivariable analysis of the secondary endpoint.

	**30-day mortality**			
**Variables**	**HR (95% CI)**	***p*-value**	**HR (95% CI)**	***p*-value**
dHb12h (g/dl)	1.16 (1.02–1.31)	0.025	-	
dHb12h group	-		1.97 (1.16–3.34)	0.011
Age (years)	1.03 (1.01–1.04)	0.001	1.03 (1.01–1.04)	0.001
Number of shocks applied	1.01 (0.99–1.02)	0.091	1.01 (1.00–1.02)	0.043
Initial shockable rhythm	0.90 (0.83–0.97)	0.005	0.90 (0.84–0.97)	0.007
Total dose of adrenaline (mg)	1.23 (1.12–1.35)	<0.001	1.22 (1.11–1.34)	<0.001
pH value	-	-	0.19 (0.06–0.68)	0.011

*30-day mortality was assessed by the Cox regression model using a backward stepwise elimination approach according to Wald test statistic step-by-step. The presented variables are the ones remaining in the final step of the model*.

*dHb12h (g/dl) = Hemoglobin (Hb) levels 12 h after return of spontaneous circulation (ROSC)—Hb levels on admission. dHb12h group = group A (equals a decrease in Hb concentrations of > 1g/dL in 12 h after ROSC) vs. group B (equals any increase in Hb or a decrease of ≤ 1 g/dL in 12 h after ROSC)*.

*CI, confidence interval; Hb, hemoglobin; HR, hazard ratio*.

In univariate analysis, the crude HR of the Hb increase group for 30-day mortality was 2.06 (95% CI 1.22–3.48, *p* = 0.007). After multivariable adjustment for the above-mentioned covariates, the estimates for the Hb increase category on 30-day mortality showed a HR of 1.97 (95% CI 1.16–3.34, *p* = 0.011) (Table 4). Patients in group B have an ~2-fold increased risk to die within 30-day after cardiopulmonary resuscitation (CPR).

**Figure 3:**
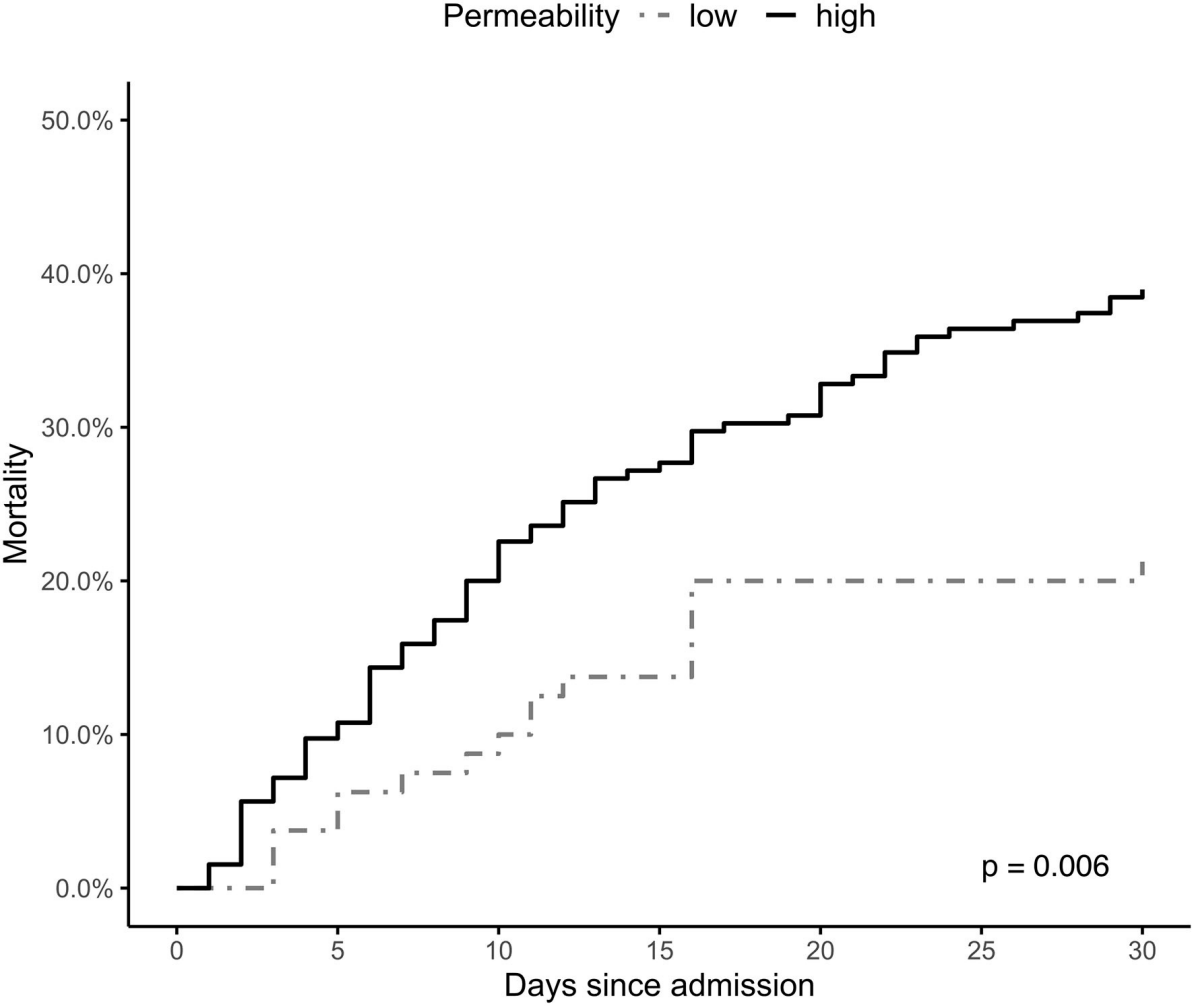
Hb-stratified Kaplan-Meier estimates of the probability of 30-day mortality among patients successfully resuscitated from cardiac arrest. Group A = a decrease in hemoglobin (Hb) levels >1 g/dL within 12 h after return of spontaneous circulation (ROSC); Group B = increase in Hb levels or a decrease of <1 g/dL Hb within 12 h after ROSC.

The Kaplan-Meier survival plot of the estimated 30-day mortality according to both groups is presented in Figure 3. The between-group difference continues throughout the observation time and is still observed on day 30 (log-rank test *p* = 0.006).

### Exploratory Analyses

Although there was a significant difference between patients with good or poor neurologic outcome in dHb12h [−0.5 g/dL (IQR: −1.4–0.5) vs. 0.2 g/dL (IQR: −0.8–1), *p* < 0.001], overall fluid balance [1.60 L (IQR: 0.86–2.35) vs. 2.15 L (IQR: 1.20–3.00), *p* = 0.001], noradrenaline dose [0.08 mcg/kg/min (IQR: 0.04–0.17) vs. 0.15 mcg/kg/min (IQR: 0.07–0.33), *p* = < 0.001] and total urine output in the first 12 h [1.13 L (IQR: 0.60–0.17) vs. 0.75 L (IQR: 0.37–0.13), *p* < 0.001], there was no difference in the total amount of infused fluids [2.90 L (IQR: 2.15–3.65) vs. 3.03 L (IQR: 2.20–4.00, *p* = 0.32)].

DHb12h correlated poorly with noradrenaline dose (*r* = 0.19, *p* = 0.001), but not with fluid balance, urine volume, total infused volume or CPR-related variables.

**Figure 4:**
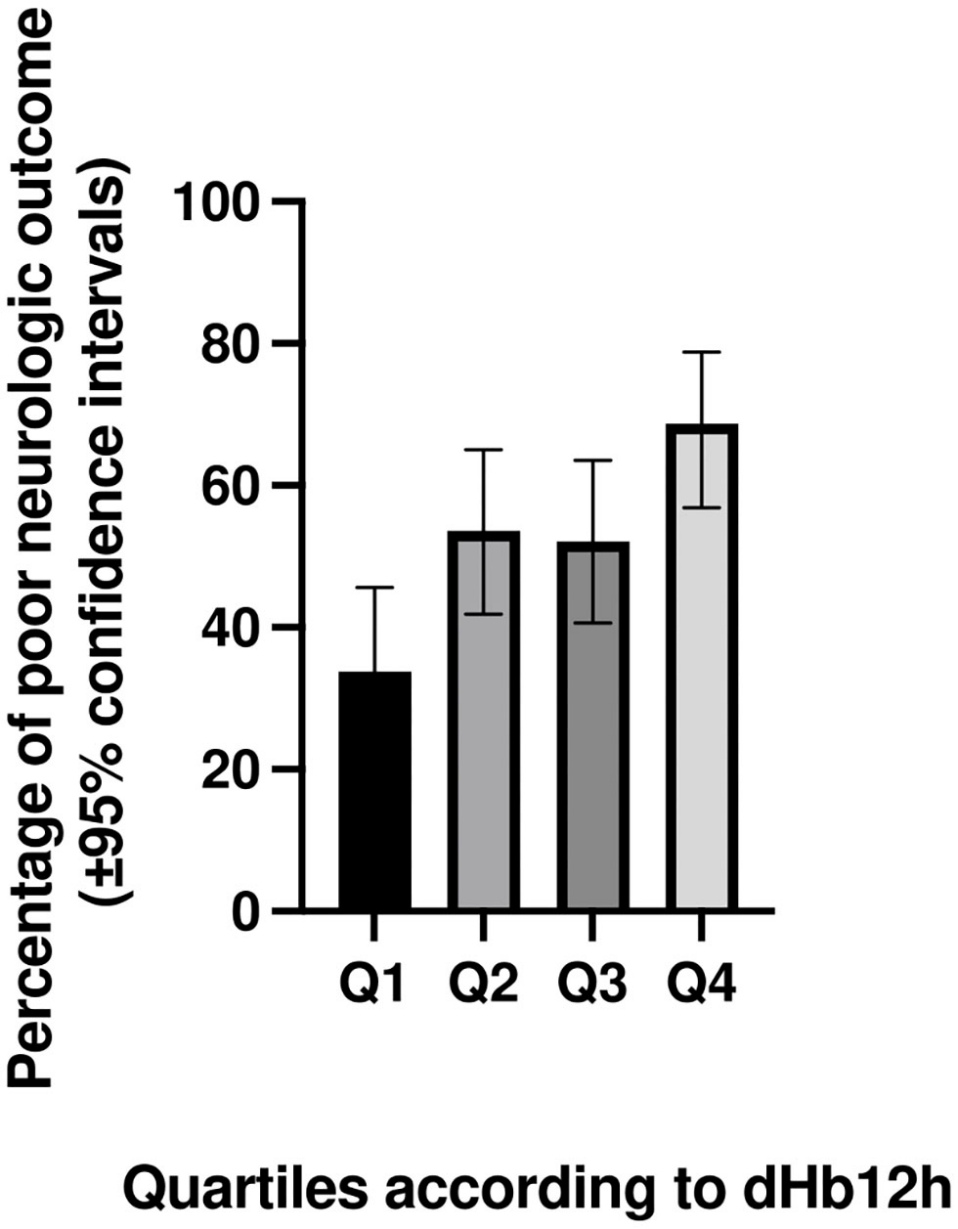
Percentage (with 95% confidence intervals) of poor neurologic function at day 30 according to dHb12h quartiles in patients successfully resuscitated from cardiac arrest. dHb12h = Hemoglobin (Hb) levels 12 h after return of spontaneous circulation—Hb levels on admission.

Table 5 shows neurologic outcome at day 30 according to dHb12h quartiles. The proportion of patients with poor neurologic function at day 30 increases from 34% in quartile 1–69% in quartile 4. The percentage of poor neurologic function at day 30 according to dHb12h quartiles is presented in Figure 4.

**Table 5:** Neurologic function at day 30 according to dHb12h quartiles.

	**Quartile 1**	**Quartile 2**	**Quartile 3**	**Quartile 3**
Good neurologic outcome, *n* (%)	45 (66)	32 (46)	34 (48)	21 (31)
Poor neurologic outcome, *n* (%)	23 (34)	37 (54)	37 (52)	46 (69)

## Discussion

The main finding of this study is that patients with increasing Hb concentrations in spite of positive fluid balance in the first 12 h after ROSC are more likely to have a poor neurologic outcome and to die within 30 days.

To our knowledge this is the first analysis of Hb kinetics in cardiac arrest survivors not focusing on Hb thresholds but on changes of Hb concentrations over time. Previous studies analyzing Hb concentrations after cardiac arrest mostly focused on the oxygen-transport capacity of Hb and associated venous oxygen saturation aiming to define a minimum Hb concentration to optimize clinical outcomes (1–5, 28–30). While Hb levels after ROSC may be associated with brain oxygenation, our study investigated Hb concentrations from a different perspective. We utilized the large size of erythrocytes, which cannot extravasate in spite of massively increased vascular permeability, and used dynamic changes in Hb concentrations over a 24 h period after resuscitation as a surrogate parameter for the degree of vascular permeability, as suggested by data from capillary leak syndrome or burn injury (8–12, 17, 18). Increased permeability may reflect the extent of post-cardiac arrest inflammation and may be associated with cerebral edema and dysfunction. In this context, results of our study showed that group B had an adjusted OR of 2.7 to have poor neurologic outcome.

Owing to the retrospective nature of this analysis, we were not able to quantify biomarkers of vascular permeability to further support our hypothesis. However, several factors may contribute to increased vascular permeability after successful cardiopulmonary resuscitation and could be of interest for further clinical studies. The endothelial glycocalyx covers the luminal surface of endothelial cells and, among other functions, is a critical regulator of fluid homeostasis (31). In the interplay of reactive oxygen species, inflammatory cytokines and activated leukocytes, the endothelial glycocalyx is degraded during ischemia-reperfusion injury by specific enzymes, so-called “sheddases,” as demonstrated in various disease models and indicated by increased shedding of glycocalyx constituents after cardiac arrest (32, 33). Levels of syndecan-1 and hyaluronan measured immediately after ROSC predicted multiple organ failure and poor clinical outcome (33). In sepsis, angiopoietin-2 is considered a key protein in the regulation of vascular permeability and endothelial activation, which is underlined by clinical data that suggest associations of angiopoietin-2 levels and sepsis-associated mortality, associations of angiopoietin-2 levels with fluid overload in septic shock patients, and by animal models showing benefits of angiopoietin-2 blockade (13, 34, 35). In addition, the impact of angiopoietin-2 on vascular permeability seems to be mediated by glycocalyx degradation (36, 37). This is likewise underlined by angiopoietin-2 data from cardiac arrest patients, where imbalances in angiopoietin ratios (angiopoetin-1/angiopoetin-2) were associated with organ dysfunction and poor outcome (38). In agreement, Bro-Jeppesen et al. reported that the level of inflammation and endothelial injury, including biomarkers of degraded endothelial glycocalyx, in post-cardiac arrest patients correlated with the requirement of vasopressor support and partly also with hemodynamic variables (39). Finally, severe brain damage itself may compromise the vascular tone and thus increase vascular permeability (40).

A major advantage of using Hb kinetics as indicator for extravascular fluid loss is its prompt availability from blood gas analysis within seconds or from automatic blood cell counters within minutes. However, Hb kinetics' obvious limitation is limited specificity, as Hb concentrations are largely affected by other diseases and complications of critical illness per se. To maximize the validity of Hb kinetics in the presented study, we analyzed a homogenous patient collective. Therefore, (i) patients with signs of bleeding, receiving transfusions or requiring surgery were excluded, as these conditions may impact on Hb levels for other reasons than endothelial dysfunction; (ii) Only patients uniformly treated with TTM were included, as hypothermia per se may impact on vascular permeability (15, 16).

Due to the retrospective nature of the presented study, we can only speculate, whether increasing Hb concentrations should be counteracted by increasing fluid administration. However, increasing data suggest that especially hypervolemia is associated with poor outcome in critical illness (41). In agreement, patients with poor neurologic outcome had a greater positive fluid balance (and vasopressor requirement) in our study. Therefore, therapies targeting vascular permeability or the deterioration of endothelial function may be better suited to optimize patient outcome. The close association between endothelial dysfunction and volume resuscitation as well as their impact on organ dysfunction thereby requires optimal fluid resuscitation, guided by additional assessment, most likely echocardiography or continuous measurement of cardiac output. It needs to be determined, whether Hb kinetics may complement the evaluation of the patient's fluid status or should rather be seen as a prognostic marker.

In contrast to septic patients, where large clinical trials are available, prospective trials on fluid resuscitation (and endothelial dysfunction) in cardiac arrest are limited. In a small randomized trial (*n* = 24) Heradstveit et al. showed that infusion of hypertonic saline and hydroxyethyl starch significantly reduced the total fluid volume, improved fluid balance and increased serum osmolality compared to a standard crystalloid fluid regimen, but failed to show an impact on magnetic resonance imaging diagnosed brain oedema formation, which was, however, largely absent in their population (42). More recently, in a randomized placebo-controlled trial, iloprost, a prostacyclin analog aiming to improve endothelial function and reduce ischemia-reperfusion injury, failed to show beneficial effects in post-cardiac arrest patients (43). In a small randomized trial, urinastatin, an inhibitor of neutrophil elastase, was ineffective with regards to clinical endpoints (44). In a small randomized trial, Geri et al. attempted to remove inflammatory mediators from circulation of cardiac arrest survivors by means of high cut-off continuous veno-venous hemofiltration, but could not show effects on proinflammatory cytokines or clinical outcomes (45).

In addition, prospective studies have addressed vasopressor-complementary strategies. Two trials investigated the effects of adding vasopressin and corticosteroids to adrenaline during resuscitation itself and adding hydrocortisone to the treatment regimen in patients with post-resuscitation shock and found improved survival of these mostly in-hospital patients (46, 47). In contrast to other trials, patients received 40 mg methylprednisolone early during resuscitation, which may have had a more pronounced impact on ischemia-reperfusion injury. Furthermore, steroids provide a potent anti-inflammatory effect, which may be superior to other above-mentioned treatment strategies. Interestingly, dexamethasone increases angiopoietin-1, with antagonistic properties to angiopoietin-2, and decreases vascular endothelial growth factor, which disrupts endothelial cell junctions and increases permeability, thereby also providing an inflammation independent mechanism of improving vascular permeability (48). Moreover, a bolus infusion of steroids is practicable, inexpensive and widely available. Comparable data in out-of-hospital cardiac arrest patients, however, are lacking.

### Limitations

The main limitation of this study is its retrospective, observational single center design. Markers of endothelial damage, constituents of the endothelial glycocalyx or biomarkers of leucocyte activation have not been measured to support our findings. Our hypothesis was driven by observations and data from other diseases and other clinical scenarios, e.g., capillary leak syndrome and burn injury, and on general theoretical considerations including the size of erythrocytes and the likelihood of erythrocyte extravasation, but we acknowledge that have no experimental data to support it. Although we aimed to exclude any form of systematic bias by the application of in- and exclusion criteria and pathophysiological considerations, there is a remaining risk of systemic bias by not including further factors interfering with acute changes in hemoglobin levels after CPR. Furthermore, it needs to be noted that we analyzed a selected patient cohort, which limits the generalizability of our study results. Likewise, selection bias arising from missing data cannot be fully excluded. Thus, appropriate caution should be applied when interpreting our findings.

### Conclusions

In conclusion, extravascular fluid loss as indicated by increasing Hb levels in cardiac arrest survivors appear to be crucial for both, neurologic function and survival. The clinical value of Hb kinetics to guide fluid resuscitation needs to be determined. However, Hb kinetics could display a surrogate marker for endothelial dysfunction and vascular permeability, which could be useful for addressing the effectiveness of endothelium-targeting therapeutic interventions in patients successfully resuscitated from cardiac arrest.

## Data Availability Statement

The raw data supporting the conclusions of this article will be made available by the authors, without undue reservation.

## Ethics Statement

The studies involving human participants were reviewed and approved by Ethics Committee of the Medical University of Vienna. Written informed consent for participation was not required for this study in accordance with the national legislation and the institutional requirements.

## Author Contributions

CScho, CSchr, BJ, NB, and MS conceived the study. CSchr, MP, CC, MM, FE, IM, MB, MH, and FS participated in data acquisition and including quality control. CScho, CSchr, JG, and MS provided statistical advice on study design and analyzed the data. CSchr drafted the macuscript. CScho takes responsibility for the paper as a whole. All authors contributed substantially to its revision, read, and approved the final manuscript.

## Conflict of Interest

The authors declare that the research was conducted in the absence of any commercial or financial relationships that could be construed as a potential conflict of interest.
