# Supplementary material for: Change of Hemoglobin Levels in the Early Post-cardiac Arrest Phase Is Associated With Outcome
Source: Front Med (Lausanne). 2021 Jun 9;8:639803. doi: 10.3389/fmed.2021.639803 (PMC8219926; doi:10.3389/fmed.2021.639803)
Supplement: Supplementary file 1 [file Data_Sheet_1.pdf]

Supplementary Material

1 Receiver-operating characteristic curve results

To identify the optimal cut-off for the dichotomization of the population we performed receiver-operator characteristic curve analyses. Several cutoffs for the primary were analyzed, as follows:

-2g: 0.52 (95% Confidence Intervals: 0.46 - 0.59, $p = 0.5$) Sensitivity: 92% Specificity: 13%

-1g: 0.59 (95% CI: 0.52 - 0.66, $p = 0.009$) Sensitivity: 80% Specificity: 39%

-0.5g: 0.58 (95% CI: 0.52 - 0.65, $p = 0.019$) Sensitivity: 66% Specificity: 51%

0g: 0.60 (95% CI: 0.53 - 0.67 $p = 0.004$) Sensitivity: 52% Specificity: 68%

0.5g: 0.59 (95% CI: 0.53 - 0.66: $p = 0.009$) Sensitivity: 39% Specificity 79%

1g: 0.54 (95% CI: 0.48 - 0.61, $p = 0.21$) Sensitivity 20% Specificity 89%

The receiver operating characteristic curve of the continuous variable dHb12h did not provide a clear cutoff (figure 2). Thus, we analyzed several cutoffs and found similar areas under the curves. Therefore, we based our decision to use -1g/dL as cutoff on a high sensitivity of 80% and an acceptable specificity of 39%. Furthermore, this cutoff shows reasonable performance also for the secondary endpoint 30-day mortality.

2 Sensitivity analysis for out-of-hospital cardiac arrest

Sensitivity analysis of the primary endpoint in patients with out-of-hospital cardiac arrest (n=255) only.

In univariate analysis, the crude OR for dHb12h was 1.23 (95% CI, 1.03 - 1.45, *p* = 0.019) to have poor neurologic outcome. After multivariable adjustment for age, sex, initial rhythm, basic life support, witness status, number of shocks, cumulative adrenaline (epinephrine) dose, pH on admission, fluid balance and noradrenaline (norepinephrine) dose the adjusted OR of dHb12h was 1.29 (95% CI, 1.05 - 1.58, *p* = 0.018) to have a poor neurologic outcome (Table S1). These results correspond to an approximate 23%, respectively 29%, increased odds of poor outcome for every g/dL increase in Hb concentrations within the first 12 hours after ROSC.

The presented variables are the ones remaining in the final step of the model:

	**Poor outcome (Cerebral Performance Category 3-5)**	
Variables	**OR (95% CI)**	p-value
dHb12h [g/dl]	1.29 (1.05-1.58)	0.018
Age [years]	1.03 (1.01-1.05)	0.002
Sex	0.56 (0.29-1.09)	0.089
Initial shockable rhythm	1.06 (0.99-1.13)	0.094
Number of shocks applied	0.78 (0.69-0.87)	<0.001
Total dose of adrenaline [mg]	1.83 (1.49-2.24)	<0.001
Fluid balance 12h [L]	1.30 (1.03-1.65)	<0.001

Table S1
dHb12h [g/dl] = Hb levels 12h after ROSC – Hb levels on admission.

In univariate analysis, the crude OR for group B was 2.30 (95% CI, 1.33 - 3.97, *p* = 0.003) to have poor neurologic outcome. After multivariable adjustment for age, sex, initial rhythm, basic life support, witness status, number of shocks, cumulative epinephrine dose, pH on admission, fluid balance and norepinephrine dose the adjusted OR of group B was 2.70 (95% CI, 1.38 - 5.29, *p* = 0.004) to have a poor neurologic outcome (Table S2). These results correspond to an approximate 2.3-fold, respectively 2.7-fold increased odds of poor outcome for group B.

The presented variables are the ones remaining in the final step of the model:

Variables	Poor outcome (Cerebral Performance Category 3-5) OR (95% CI)	p-value
dHb12h group	2.70 (1.38-5.29)	0.004
Age [years]	1.03 (1.01-1.05)	0.001
Initial shockable rhythm	1.05 (0.98-1.13)	0.133
Number of shocks applied	0.79 (0.71-0.89)	<0.001
Total dose of epinephrine [mg]	1.68 (1.35-2.09)	<0.001
pH	0.17 (0.02-1.62)	0.124
Fluid balance 12h [L]	1.31 (1.03-1.66)	0.03

Table S2
dHb12h group = group A (equals a decrease in Hb concentrations of >1g/dL in 12h after ROSC) vs. group B (equals any increase in Hb or a decrease of ≤1g/dL in 12h after ROSC)

3 Sensitivity analysis for basic life support

Sensitivity analysis of the primary endpoint in patients with basic life support (n=175).

In univariate analysis, the crude OR for dHb12h was 1.12 (95% CI, 0.91 - 1.38, $p = 0.28$) to have poor neurologic outcome. In multivariable analysis, the variable dHb12h was eliminated from the model.

In univariate analysis, the crude OR for group B was 2.38 (95% CI, 1.24 – 4.56, $p = 0.009$) to have poor neurologic outcome. After multivariable adjustment for age, sex, initial rhythm, witness status, number of shocks, cumulative epinephrine dose, pH on admission, fluid balance and norepinephrine dose the adjusted OR of group B was 2.41 (95% CI, 1.11 – 5.25, $p = 0.027$) to have a poor neurologic outcome (Table S3). These results correspond to an approximate 2.4-fold increased odds of poor outcome for group B.

The presented variables are the ones remaining in the final step of the model:

Variables	Poor outcome (Cerebral Performance Category 3-5) OR (95% CI)	p-value
dHb12h group	2.41 (1.11-5.25)	0.027
Age [years]	1.04 (1.01-1.07)	0.001
Number of shocks applied	0.80 (0.70-0.91)	0.001
Norepinephrine dose	4.76 (0.84-26.95)	0.0078
Total dose of epinephrine [mg]	1.48 (1.17-1.87)	0.001
pH	0.04 (0.003-0.60)	0.02

Table S3
dHb12h group = group A (equals a decrease in Hb concentrations of >1g/dL in 12h after ROSC) vs. group B (equals any increase in Hb or a decrease of ≤1g/dL in 12h after ROSC)

4 Sensitivity analysis for patients without basic life support

Sensitivity analysis of the primary endpoint in patients without basic life support (n=100).

In univariate analysis, the crude OR for dHb12h was 1.50 (95%CI 1.12 - 2.0, p = 0.006) to have poor neurologic outcome. After multivariable adjustment for age, sex, initial rhythm, basic life support, witness status, number of shocks, cumulative epinephrine dose, pH on admission, fluid balance and norepinephrine dose the adjusted OR of dHb12h was 1.50 (95%CI, 1.09 – 2.07, p = 0.013) to have a poor neurologic outcome (Table S4). These results correspond to an approximate 50% increased odds of poor outcome for every g/dL increase in Hb concentrations within the first 12 hours after ROSC. The presented variables are the ones remaining in the final step of the model:

Variables	**Poor outcome (Cerebral Performance Category 3-5)** OR (95% CI)	p-value
dHb12h [g/dl]	1.50 (1.09-2.07)	0.013
Age [years]	1.03 (0.99-1.06)	0.58
Number of shocks applied	0.84 (0.73-0.98)	0.024
Total dose of epinephrine [mg]	1.72 (1.27-2.32)	<0.001

Table S4
dHb12h [g/dl] = Hb levels 12h after ROSC – Hb levels on admission.

In univariate analysis, the crude OR for group B was 2.38 (95% CI, 1.24 – 4.56, p = 0.009) to have poor neurologic outcome. After multivariable adjustment for age, sex, initial rhythm, witness status, number of shocks, cumulative epinephrine dose, pH on admission, fluid balance and norepinephrine dose the adjusted OR of the group B was 2.41 (95% CI, 1.11 – 5.25, p = 0.027) to have a poor neurologic outcome (Table S5). These results correspond to an approximate 2.4-fold increased odds of poor outcome for the group B.

The presented variables are the ones remaining in the final step of the model:

Variables	**Poor outcome (Cerebral Performance Category 3-5)** OR (95% CI)	p-value
dHb12h group	3.52 (1.04-11.95)	0.043
Age [years]	1.03 (1.0-1.06)	0.054
Number of shocks applied	0.83 (0.71-0.97)	0.016
Total dose of epinephrine [mg]	1.89 (1.36-2.63)	<0.001
Initial shockable rhythm	1.07 (0.94-1.21)	0.33

Table S5
dHb12h group = group A (equals a decrease in Hb concentrations of >1g/dL in 12h after ROSC) vs. group B (equals any increase in Hb or a decrease of ≤1g/dL in 12h after ROSC)

5 Sensitivity analysis for expected hemodilution vs. observed hemoglobin concentrations

In a first step we calculated the estimated total blood volume of each patient according to the Nadler Equation

Men: (0.3699 + height^3) + (0.03219*weight) + 0.6041
Women: (0.3561*height^3) + (0.03308*weight) + 0.1833

We then calculated a hemodilution factor:

Hemodilution Factor = (Total blood volume + 12h fluid balance) / Total blood volume

In the next step we calculated the expected hemoglobin concentrations by division of the hemodilution factor:

Expected hemoglobin at 12h = hemoglobin concentration at admission / hemodilution factor.

We then calculated differences between actual hemoglobin concentrations at 12h and the expected value. This value is termed "diffHb_12h" in the following.

In receiver-operating characteristic curves, the AUC of this new variable was 0.65 (95% CI 0.59 - 0.72, $p < 0.001$).

We included this variable in our logistic regression model. In univariate analysis, the crude OR of diffHb_12h was 1.27 (95% CI 1.13 - 1.44, $p < 0.001$) to have a poor neurologic outcome. After multivariable adjustment for age, sex, initial rhythm, basic life support, witness status, number of shocks, cumulative epinephrine dose, pH on admission, fluid balance and norepinephrine dose the adjusted OR of diffHb_12h was 1.19 (95% CI, 1.04 - 1.37, $p = 0.009$) to have a poor neurologic outcome (Table S4). These findings indicate an increase of 27%, respectively 19%, in the odds to have poor neurologic outcome for each g/dL difference between the expected and the actual hemoglobin concentration 12h after admission.

Variables	**Poor outcome (Cerebral Performance Category 3-5)** **OR (95% CI)**	p-value
DiffHb_12h	1.19 (1.04-1.37)	0.009
Age [years]	1.04 (1.02-1.07)	<0.001
Sex	0.55 (0.29-1.05)	0.007
Number of shocks applied	0.81 (0.73-0.90)	<0.001
Total dose of epinephrine [mg]	1.55 (1.28-1.88)	<0.001
pH	0.07 (0.01-0.58)	0.015

Table S6
DiffHb_12h = difference between expected and measured hemoglobin concentrations.

Limitations of this model: Also in healthy individuals only part of crystalloid solutions remain within the circulation and the rest diffuses to extravascular tissues. This was neglected in this analysis. The total blood volume was calculated based on the formula of Nadler et al., which naturally is only an approximation, but not a precise measurement.

6 Sensitivity analysis with inflammatory biomarkers

We included C-reactive protein concentrations and white blood cell counts at admission in the multivariable analysis of the primary endpoint. Both parameters were eliminated from the model, and the results remained stable with an OR of dHb12h of 1.28 (95%CI, 1.05 - 1.56, *p* = 0.014).

Variables	**Poor outcome (Cerebral Performance Category 3-5)** **OR (95% CI)**	p-value
dHb12h [g/dl]	1.28 (1.05-1.56)	0.014
Age [years]	1.04 (1.02-1.06)	0.001
Initial shockable rhythm	1.05 (0.99–1.13)	0.131
Number of shocks applied	0.81 (0.73-0.90)	<0.001
Total dose of epinephrine [mg]	1.57 (1.29-1.91)	<0.001
pH	0.13 (0.02-1.12)	0.063
Fluid Balance at 12h	1.27 (1.02-1.60)	0.035

Table S7
dHb12h [g/dl] = Hb levels 12h after ROSC – Hb levels on admission.

Furthermore, we included C-reactive protein concentrations at 48 hours, white blood cell count at 48 hours and 24-hours fluid balance (instead of 12-hours fluid balance) in the multivariable analysis of the primary endpoint.

After multivariable adjustment for age, sex, initial rhythm, basic life support, witness status, number of shocks, cumulative epinephrine dose, pH on admission, 24-hours fluid balance, C-reactive protein concentrations at 48 hours and white blood cell count at 48 hours the adjusted OR of dHb12h was 1.22 (95%CI, 1.01 – 1.47, *p* = 0.044) to have a poor neurologic outcome (Table S8). C-reactive protein levels and white blood cell counts at 48 hours remained in the model.
The presented variables are the ones remaining in the final step of the model:

Variables	Poor outcome (Cerebral Performance Category 3-5) OR (95% CI)	p-value
dHb12h [g/dl]	1.22 (1.01-1.47)	0.044
Age [years]	1.03 (1.01-1.05)	0.002
Number of shocks applied	0.78 (0.69-0.87)	<0.001
Total dose of epinephrine [mg]	1.69 (1.40-2.03)	<0.001
C-reactive protein concentrations at 48 hours [mg/dl]	1.05 (1.02-10.9)	0.005
White blood cell count at 48 hours [G/l]	1.09 (1.03-1.17)	0.006

Table S8
dHb12h [g/dl] = Hb levels 12h after ROSC – Hb levels on admission.

7 Multivariable analysis of the primary endpoint for dHB12h as continuous variable

Poor outcome was analyzed by binary logistic regression using a backward stepwise elimination approach according to Wald test statistic step-by-step. All steps of the procedure are presented in the following:

Variables in the Equation

		B	S.E.	Wald	df	Sig.	Exp(B)	95% C.I.for EXP(B)	
								Lower	Upper
Step 1[a]	Age	,035	,010	11,782	1	,001	1,036	1,015	1,057
	Sex	-,438	,332	1,740	1	,187	,645	,336	1,237
	Shockable Rhythm	,053	,036	2,189	1	,139	1,055	,983	1,131
	Witnessed	-,002	,005	,106	1	,745	,998	,988	1,009
	BLS	-,018	,318	,003	1	,955	,982	,526	1,833
	Number of Shocks	-,204	,054	14,266	1	,000	,815	,733	,906
	Epinephrine dose	,449	,100	20,271	1	,000	1,566	1,288	1,904

	pH	-1,891	1,108	2,912	1	,088	,151	,017	1,324
	dHb12h	,243	,104	5,517	1	,019	1,276	1,041	1,563
	Fluid Balance 12h	,204	,122	2,786	1	,095	1,226	,965	1,557
	Norepinephrine dose	,649	,630	1,059	1	,303	1,913	,556	6,583
	Constant	10,897	7,856	1,924	1	,165	54022,308		
Step 2[a]	Age	,035	,010	11,779	1	,001	1,036	1,015	1,057
	Sex	-,437	,331	1,739	1	,187	,646	,338	1,237
	Shockable Rhythm	,053	,036	2,209	1	,137	1,055	,983	1,131
	Witnessed	-,002	,005	,102	1	,749	,998	,988	1,009
	Number of Shocks	-,205	,054	14,616	1	,000	,815	,734	,905
	Epinephrine dose	,449	,099	20,385	1	,000	1,567	1,289	1,904
	pH	-1,892	1,108	2,919	1	,088	,151	,017	1,321
	dHb12h	,244	,103	5,615	1	,018	1,276	1,043	1,562
	Fluid Balance 12h	,204	,122	2,807	1	,094	1,226	,966	1,557
	Norepinephrine dose	,651	,629	1,069	1	,301	1,917	,558	6,580
	Constant	10,897	7,857	1,924	1	,165	54014,454		
Step 3[a]	Age	,035	,010	11,757	1	,001	1,036	1,015	1,057
	Sex	-,433	,331	1,714	1	,190	,648	,339	1,240
	Shockable Rhythm	,053	,036	2,214	1	,137	1,055	,983	1,131
	Number of Shocks	-,204	,053	14,657	1	,000	,815	,734	,905
	Epinephrine dose	,448	,099	20,355	1	,000	1,564	1,288	1,900

	pH	-1,911	1,104	2,995	1	,084	,148	,017	1,288
	dHb12h	,243	,103	5,580	1	,018	1,275	1,042	1,559
	Fluid Balance 12h	,204	,122	2,800	1	,094	1,226	,966	1,557
	Norepinephrine dose	,635	,625	1,029	1	,310	1,886	,554	6,426
	Constant	11,021	7,833	1,980	1	,159	61169,129		
Step 4[a]	Age	,036	,010	12,308	1	,000	1,036	1,016	1,057
	Sex	-,494	,325	2,302	1	,129	,610	,323	1,155
	Shockable Rhythm	,053	,035	2,355	1	,125	1,055	,985	1,129
	Number of Shocks	-,207	,053	15,008	1	,000	,813	,733	,903
	Epinephrine dose	,456	,099	21,393	1	,000	1,578	1,300	1,914
	pH	-2,093	1,084	3,731	1	,053	,123	,015	1,031
	dHb12h	,255	,102	6,220	1	,013	1,290	1,056	1,576
	Fluid Balance 12h	,243	,115	4,442	1	,035	1,275	1,017	1,599
	Constant	12,376	7,675	2,600	1	,107	237082,055		
Step 5[a]	Age	,035	,010	11,787	1	,001	1,035	1,015	1,056
	Shockable Rhythm	,052	,034	2,277	1	,131	1,053	,985	1,126
	Number of Shocks	-,208	,053	15,469	1	,000	,812	,732	,901
	Epinephrine dose	,452	,099	20,737	1	,000	1,572	1,294	1,910
	pH	-2,011	1,083	3,448	1	,063	,134	,016	1,118
	dHb12h	,248	,101	6,030	1	,014	1,281	1,051	1,561
	Fluid Balance 12h	,242	,115	4,437	1	,035	1,274	1,017	1,595

		B	S.E.	Wald	df	Sig.	Exp(B)	Lower	Upper
	Constant	11,506	7,664	2,254	1	,133	99312,573		
Step 6[a]	age	,035	,010	12,283	1	,000	1,036	1,015	1,056
	Number of Shocks	-,198	,052	14,753	1	,000	,820	,741	,908
	Epinephrine dose	,437	,096	20,717	1	,000	1,548	1,283	1,869
	pH	-2,185	1,076	4,126	1	,042	,112	,014	,926
	dHb12h	,240	,098	6,005	1	,014	1,272	1,049	1,541
	Fluid Balance 12h	,206	,112	3,353	1	,067	1,228	,986	1,531
	Constant	12,900	7,606	2,877	1	,090	400396,368		

Table S9

8 Multivariable analysis of the primary endpoint for dHB12h categories

Poor outcome was analyzed by binary logistic regression using a backward stepwise elimination approach according to Wald test statistic step-by-step. All steps of the procedure are presented in the following:

Variables in the Equation

								95% C.I.for EXP(B)	
		B	S.E.	Wald	df	Sig.	Exp(B)	Lower	Upper
Step 1[a]	Age	,036	,010	11,937	1	,001	1,036	1,016	1,057
	Sex	-,417	,333	1,566	1	,211	,659	,343	1,266
	Shockable Rhythm	,052	,036	2,055	1	,152	1,054	,981	1,132
	Witnessed	-,001	,005	,046	1	,831	,999	,988	1,010
	BLS	-,014	,317	,002	1	,965	,986	,530	1,835

	Number of Shocks	-,195	,054	13,188	1	,000	,822	,740	,914
	Epinephrine Dose	,442	,099	19,810	1	,000	1,556	1,281	1,890
	pH	-2,086	1,117	3,487	1	,062	,124	,014	1,109
	Fluid Balance 12h	,212	,123	2,963	1	,085	1,236	,971	1,573
	Norepinephrine Dose	,577	,635	,825	1	,364	1,781	,513	6,188
	Group B	,974	,338	8,328	1	,004	2,650	1,367	5,136
	Constant	11,483	7,892	2,117	1	,146	97065,374		
Step 2[a]	Age	,036	,010	11,935	1	,001	1,036	1,016	1,057
	Sex	-,416	,332	1,568	1	,210	,660	,344	1,265
	Shockable Rhythm	,052	,036	2,067	1	,151	1,054	,981	1,132
	Witnessed	-,001	,005	,044	1	,834	,999	,988	1,010
	Number of Shocks	-,196	,053	13,467	1	,000	,822	,741	,913
	Epinephrine Dose	,442	,099	19,915	1	,000	1,556	1,282	1,890
	pH	-2,087	1,116	3,494	1	,062	,124	,014	1,107
	Fluid Balance 12h	,212	,123	2,987	1	,084	1,236	,972	1,573
	Norepinephrine dose	,579	,634	,832	1	,362	1,783	,514	6,185
	Group B	,976	,336	8,430	1	,004	2,653	1,373	5,127
	Constant	11,482	7,893	2,116	1	,146	96944,315		
Step 3[a]	Age	,036	,010	11,939	1	,001	1,036	1,015	1,057
	Sex	-,413	,332	1,554	1	,213	,661	,345	1,267
	Shockable Rhythm	,052	,036	2,072	1	,150	1,054	,981	1,132

	Number of Shocks	-,196	,053	13,497	1	,000	,822	,741	,913
	Epinephrine Dose	,442	,099	19,901	1	,000	1,555	1,281	1,889
	pH	-2,099	1,114	3,552	1	,059	,123	,014	1,087
	Fluid Balance 12h	,213	,123	2,998	1	,083	1,237	,972	1,574
	Norepinephrine Dose	,566	,630	,807	1	,369	1,761	,512	6,057
	Group B	,977	,336	8,445	1	,004	2,656	1,374	5,131
	Constant	11,562	7,875	2,155	1	,142	104989,003		
Step 4[a]	Age	,036	,010	12,416	1	,000	1,037	1,016	1,058
	Sex	-,464	,327	2,016	1	,156	,629	,331	1,193
	Shockable Rhythm	,052	,035	2,179	1	,140	1,054	,983	1,129
	Number of Shocks	-,197	,053	13,728	1	,000	,821	,740	,911
	Epinephrine Dose	,449	,098	20,862	1	,000	1,567	1,292	1,901
	pH	-2,279	1,092	4,361	1	,037	,102	,012	,869
	Fluid Balance 12h	,247	,116	4,512	1	,034	1,280	1,019	1,608
	Group B	1,017	,333	9,319	1	,002	2,765	1,439	5,312
	Constant	12,864	7,711	2,783	1	,095	386155,313		
Step 5[a]	Age	,035	,010	11,812	1	,001	1,036	1,015	1,056
	Shockable Rhythm	,051	,035	2,127	1	,145	1,052	,983	1,126
	Number of Shocks	-,199	,053	14,158	1	,000	,820	,739	,909
	Epinephrine Dose	,445	,099	20,202	1	,000	1,561	1,285	1,895
	pH	-2,201	1,092	4,062	1	,044	,111	,013	,941

	Fluid Balance 12h	,249	,116	4,636	1	,031	1,283	1,023	1,609
	Group B	1,017	,332	9,402	1	,002	2,765	1,443	5,296
	Constant	12,045	7,704	2,444	1	,118	170311,002		
Step 6[a]	Age	,035	,010	12,312	1	,000	1,036	1,016	1,056
	Number of Shocks	-,189	,052	13,448	1	,000	,828	,748	,916
	Epinephrine Dose	,430	,096	20,259	1	,000	1,537	1,275	1,854
	pH	-2,401	1,083	4,918	1	,027	,091	,011	,757
	Fluid Balance 12h	,210	,113	3,477	1	,062	1,234	,989	1,539
	Group B	,990	,326	9,254	1	,002	2,692	1,422	5,096
	Constant	13,658	7,635	3,200	1	,074	853905,254		

Table S10
